# Construction of Pd Single Site Anchored on Nitrogen-Doped Porous Carbon and Its Application for Total Antioxidant Level Detection

**DOI:** 10.1186/s11671-022-03693-5

**Published:** 2022-05-20

**Authors:** Jingwen Zhang, Zhi Li, Hui Li, Ge Dai, Feifei Luo, Zhaohui Chu, Xing Geng, Fan Zhang, Qingjiang Wang

**Affiliations:** 1 School of Chemistry and Molecular Engineering, East China Normal University, 500 Dongchuan Road, Shanghai, 200241 People’s Republic of China; 2 School of Chemistry and Chemical Engineering, Shanghai Jiaotong University, 800 Dongchuan Road, Shanghai, 200240 People’s Republic of China

**Keywords:** Mimic enzymes, Single-atomic palladium, Porous material, Glutathione, Ascorbic acid

## Abstract

Natural enzymes have excellent catalytic activity. However, due to their unstable nature and high cost, current research has turned to the synthesis and development of enzyme-like nanomaterials and single-atomic nanozymes. In this study, a single-atomic palladium-loaded nitrogen-doped porous carbon catalyst (SA-Pd/NPC) was prepared and used as a mimetic peroxidase to catalyze the substrates oxidation. The catalytic capability of the SA-Pd/NPC was tested by the TMB-H_2_O_2_ system, and it expressed a superior catalytic capability owing to the plentiful catalytic centers of the single-atom Pd, its high porosity, the large specific surface area, and the strong electron transfer capability of the NPC. For the color reaction of TMB, thiol antioxidants (e.g., glutathione, GSH) and non-thiol antioxidants (e.g., ascorbic acid, AA) are suitable for different inhibition mechanisms. GSH and AA are typical substances of these two main antioxidant types, respectively. Here, we demonstrate that this prepared catalyst could be used to simultaneously determine a variety of major known physiologically relevant thiol-containing and thiol-free antioxidants, accompanied by a blue color gradient change with UV–Vis spectra at 652 nm through the SA-Pd/NPC-catalyzed TMB-H_2_O_2_ system. Linear responses to GSH and AA could be obtained in the concentration ranges of 0.01–0.10 mM and 1–13 μM (both R^2^ values were greater than 0.970), respectively, while the limits of detection were 3 μM and 0.3 μM, respectively. The ability of the nanozyme to detect overall antioxidant levels (TAL) was also confirmed in subsequent tests on artificial saliva and biological samples.

**Supplementary Information:**

The online version contains supplementary material available at 10.1186/s11671-022-03693-5.

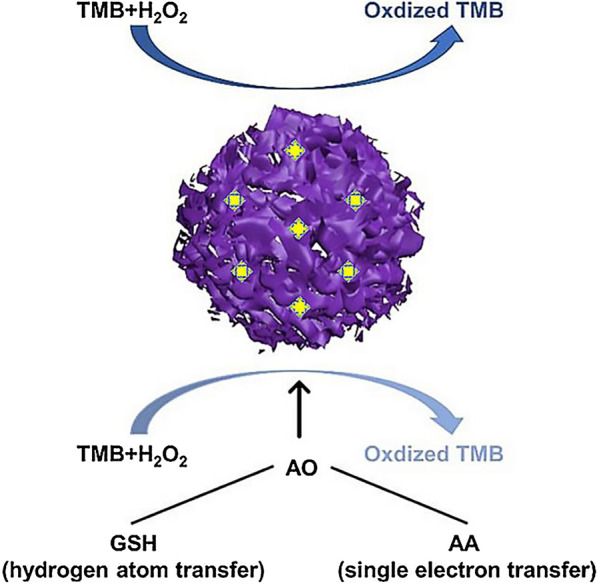

## Introduction

Enzymes are widely used in various fields such as clinical medicine, food, and environment [1, 2]. However, most natural enzymes are easily denatured and inactivated, and their conditions of use are usually rigorous [3]. Artificial mimetic enzymes with biocatalytic activities have attracted considerable attention because of their adjustable structure, high stability, low cost, and large production capability. They have a broad potential to replace natural enzymes in biological fields and nanotechnologies [4–6].

As a platinum group element, palladium is widely used as a selective and prospective catalyst for many complex chemical reactions [7]. Platinum group nanoparticles have been reported to have enzyme-like activities to catalyze the TMB-H_2_O_2_ system [8–14]. Up to now, palladium nanozymes catalyzing TMB-H_2_O_2_ systems have been studied for the detection of glucose [15], hydrogen peroxide [16], carcinoembryonic antigen [17] and heparin [10]. When the catalytically active metal atoms were loaded on the surface of nanomaterials, the maximum atom utilization could be achieved, and finally, high-performance catalysis could be realized [18, 19]. Recent research has increasingly focused on single-atom catalysts with simulated peroxidase activity [20–23].

Recently, single-atom catalysts with precise active sites have received extensive attention. For example, boron-doped Fe–N–C single-atom catalysts could be used for the sensitive detection of acetylcholinesterase activity and corresponding pesticides [20]. Fe–N–C single-atom nanozymes could be utilized for the sensing of galactose, intracellular hydrogen peroxide detection, and acetylcholinesterase (AchE) determination in human serum samples [21–23]. It was also reported that single-atomic palladium had an enzyme-like activity (called Pd SAzyme) and could be used to catalyze Fenton and Fenton-like reactions. For instance, the Pd SAzyme with an atom-economical utilization of catalytic centers could help support the removal of heat shock proteins and mediate mild temperature photothermal therapy, which simultaneously maximized the photothermal therapy efficiency and minimized damage to healthy tissues [24]. At present, single-atom Pd nanozymes have been demonstrated to have oxidase-like activities, and have been used to detect the total antioxidant capacity of fruits, determine serum acid phosphatase activity, and construct NAND logic gates [25]. However, the Pd SAzyme as a mimic peroxidase in biomarker detection is relatively rare and is still required.

Oxidative stress refers to the imbalance between oxidants and antioxidants in the body due to endogenous or exogenous stimuli [26]. Antioxidants, as a sort of free radical scavengers, can offset the adverse effects of oxidative stress and are essential for the body's redox homeostasis [27]. Antioxidants could be divided into two categories, thiol antioxidants and non-thiol antioxidants, according to whether they contain sulfhydryl groups, which combine with hydroxyl radicals according to different reaction mechanisms and prevent the color reaction of TMB to oxTMB in the presence of hydrogen peroxide [28, 29]. Glutathione (GSH) and ascorbic acid (AA) are representatives of the two types of antioxidants in the human body, which react with hydroxyl radicals through hydrogen atom transfer and single electron transfer mechanisms, respectively [29]. Simultaneously detecting these two types of antioxidants deserves further studies. The evaluation of total antioxidant levels (TAL), such as in food or biological fluids, plays a great role in human health monitoring and disease treatment [30, 31]. The development of highly sensitive and low-cost methods for assessing TAL has received widespread attention [32, 33]. The colorimetric method based on nanomaterials, due to its fast, convenient and low-cost nature, has been considered to have the potential to replace traditional methods [34].

In this work, a single-atomic palladium-loaded nitrogen-doped porous carbon catalyst (SA-Pd/NPC) was prepared and used as a Pd SAzyme for the colorimetric detection of TAL. For the synthesis steps, the aniline monomers were self-polymerized on hard silica sphere templates and then agglomerated to form a nitrogen-doped porous carbon shell. The single-site Pd could be produced by its insertion in the nitrogen caves of NPC by the Lewis acid doping strategy. As tested by the TMB-H_2_O_2_ system, the Pd SAzyme has a peroxidase-like activity and generates hydroxyl radicals on its surface. The excellent catalytic capability of Pd SAzyme is due to the plentiful catalytic centers of the single-atomic Pd and its high porosity, large specific surface area, and strong electron transfer capability of the NPC material. The overall antioxidant levels can be sensitively determined by UV–Vis spectra at 652 nm through the SA-Pd/NPC-catalyzed TMB-H_2_O_2_ system. In this paper, GSH and AA are used as typical representatives of the two antioxidant types to construct a colorimetric detection system. Compared to most TAL assays that used only ascorbic acid as a marker, the sensitive detection of these two antioxidant types was achieved and finally applied to the detection of artificial simulated saliva and biological samples. Through different experiments and calculations, the peroxidase-like activity and substrate affinity of Pd SAzyme were explored. More importantly, this Pd SAzyme can realize the simultaneous detection of two different types of antioxidants. Our results will aid further explorations of the mechanism of antioxidant mechanisms and pave the way for the effective design of more enzyme-like catalysts.

## Experimental Section

### Reagents and Materials

Hydride chloride (37 weight percent, wt%), ethanol, nitromethane, sodium hydroxide, glacial acetic acid, hydrogen peroxide solution (30 wt%), L-ascorbic acid (AA), uric acid (UA), L-cysteine (Cys), and glutathione (GSH) were purchased from Sinopharm Chemical Reagent Co. Ltd. (Shanghai, China, www.en.reagent.com.cn). Sodium acetate anhydrous was purchased from Sigma-Aldrich Chemical Co., Ltd. (St. Louis, MO, USA, www.sigmaaldrich.com/united-states.html). 2-(tert-butoxycarbonyl)-2-methyl-3,4-dihydro-2H-pyrrole 1-oxide (BMPO) was purchased from ApexBio Technology. (7505 Fannin street, Suite 410, Houston, USA, www.apexbt.com). Pd(CH_3_CN)_2_Cl_2_, ammonium hydroxide solution (25–28 wt%), 3,3′,5,5′-tetramethylbenzidine (TMB), silicon (IV) oxide, ammonium persulfate (APS), aniline, dimethyl sulfoxide (DMSO), potassium thiocyanate (KSCN), (±)-6-hydroxy-2,5,7,8-tetramethylchromane-2-carboxylic acid and DL-homocysteine (Hcy) were purchased from Shanghai Titan Scientific Co., Ltd. (Shanghai, China, www.tansoole.com). All chemicals were used as received without any further purification.

### Apparatus and Measurements

Powder X-ray diffraction (pXRD) experiments were performed on a Bruker D8Advance diffractometer (D8Advance, Bruker, Germany) using a filtered Cu-K_α_ radiation source (λ = 1.5418 Å). High-resolution transmission electron microscopy (TEM) was obtained from a JEM-2100F microscope operated at 200 kV. High-angle annular dark-field scanning transmission electron microscopy (HAADF-STEM) images were recorded by FEI Titan Themis 200 TEM at an accelerating voltage of 200 kV. Electron paramagnetic resonance (EPR) spectra were obtained from a Bruker EMXplus 9.5/12. Nitrogen adsorption–desorption isotherms were recorded on an Autosorb-IQ instrument. Before measurements, samples were degassed at 150 °C for 2 h. UV–vis measurements were performed using a Varian Cary-50 UV–vis spectrophotometer equipped with a 1-cm path length cell.

### Preparation of SA-Pd/NPC

**Fig. 1:**
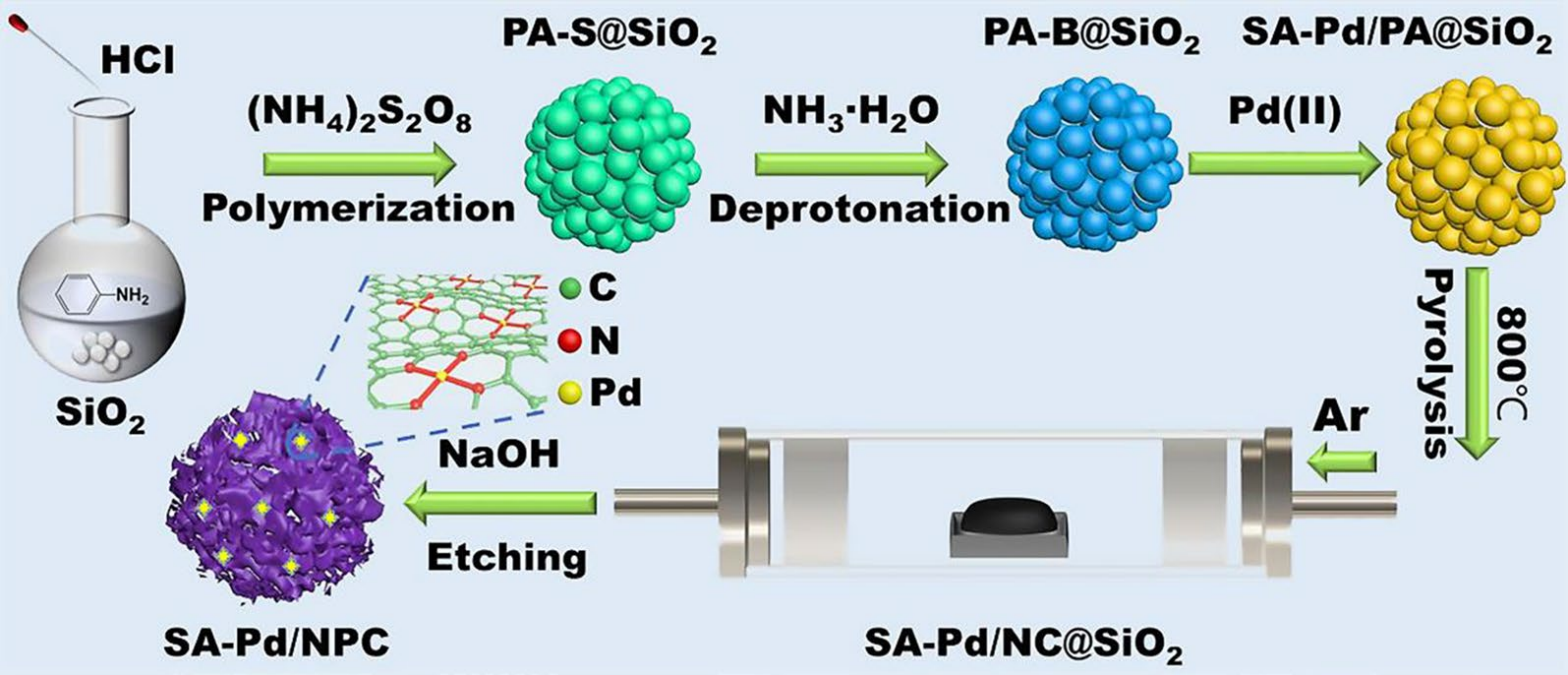
Schematic illustration of the overall synthetic procedure for SA-Pd/NPC

The synthesis procedure is shown in Fig. 1. First, colloidal silica nanoparticles (1.6 g) with a diameter of about 10 nm and aniline monomer (0.4 g) were added into 100 mL of distilled water. Then, a hydrochloric acid solution (1.2 M) was added to adjust the pH to 3 and the mixture solution was stirred for one hour. Ammonium persulfate (0.52 g) was dissolved in water (4.0 mL) and then added to the above reaction solution, which was stirred at room temperature for 15 h to carry out the polymerization reaction. After centrifugation and washing with deionized water and ethanol, an intermediate product was obtained. The product was placed into ammonia water (1 M) and stirred at room temperature for 15 h. After centrifugation, it was washed 3 times with deionized water and dried overnight at 80 °C. Subsequently, the intermediate product (450 mg), nitromethane (20 mL), and Pd (CH_3_CN)_2_Cl_2_ (0.01 mmol) were mixed and stirred at room temperature for 15 h, centrifuged and washed with nitromethane three times, and dried overnight at 80 °C. The obtained material was pyrolyzed in an argon atmosphere at 800 °C for 3 h, dissolved in 3 M NaOH aqueous solution and ethanol with a volume ratio of 1:1, and overnighted at 100 °C to remove the silica template. The final product was obtained by washing with deionized water and ethanol and drying in a vacuum at room temperature.

### Peroxidase Activity and Kinetics Assay of Pd SAzyme

Kinetic measurements of the peroxidase-like properties were confirmed by the color change of TMB, which was indicated by the absorbance at 652 nm recorded by a multimode reader. The kinetic test was detected by the change in different concentrations of TMB and H_2_O_2_. As for the kinetic assay toward TMB, the reaction was carried out at 37 °C by using 10 μg mL^−1^ of Pd SAzyme in a 1.0 mL solution containing 0.1 M H_2_O_2_ and varying TMB concentrations ranging from 41.6 μM to 0.416 mM. The HAc-NaAc (0.2 M, pH 3.5) buffer was added into the above solution until the final volume of the reaction substrate was 1.0 mL. Similarly, as for the kinetic assay toward H_2_O_2_, the reaction was carried out at 37 °C by using 10 μg mL^−1^ of Pd SAzyme in a 1.0 mL solution containing 0.416 mM TMB and varying H_2_O_2_ concentrations ranging from 5 to 100 mM. The HAc-NaAc (0.2 M, pH 3.5) buffer was added into the above solution until the final volume of the reaction substrate was 1.0 mL. Kinetic parameters were determined by fitting reaction data to the Michaelis–Menten (Eq. 1) and Lineweaver–Burk (Eq. 2) equations.

1 $$\frac{{1}}{V} = \frac{{K{\text{m}}}}{V\max [S]} + \frac{1}{{V{\text{max}}}}$$

2 $$V = \frac{{Vmax{ \times [\text{S}]}}}{{Km{ + [\text{S}]}}}$$

where *V* is the initial velocity, S is the concentration of the substrate (TMB or H_2_O_2_), *K*_m_ is the Michaelis–Menten constant, and *V*_max_ is the maximal reaction velocity.

### Detection of GSH

Glutathione (GSH) is a typical representative of sulfhydryl antioxidants, and its sensitive detection is critical in the total antioxidant performance test. 0.01–0.10 mM glutathione (GSH) aqueous solutions were, respectively, mixed with 20 μL of Pd SAzyme solution (1.0 mg mL^−1^ in ethanol), 20 μL of TMB solution (2.0 mg mL^−1^ in DMSO), and 20 μL of H_2_O_2_ solution (10 M) and diluted to 1.0 mL by 0.2 M HAc-NaAc buffer (pH 3.5). The above solutions were incubated at 37 °C for about 5 min. Finally, the linear relationship between the measured absorbance values at 652 nm and GSH concentration was used for the detection of GSH.

### Detection of AA

Ascorbic acid (AA) is a typical representative of non-sulfhydryl antioxidants, and its sensitive detection is critical in the total antioxidant performance test. First, 1–13 μM ascorbic acid (AA) aqueous solutions were, respectively, mixed with 20 μL of Pd SAzyme solution (1.0 mg mL^−1^ in ethanol), 20 μL of TMB solution (2.0 mg mL^−1^ in DMSO), and 20 μL of H_2_O_2_ solution (10 M) and diluted to 1.0 mL by 0.2 M HAc-NaAc buffer (pH 3.5). The above solutions were incubated at 37 °C for about 5 min. Finally, the linear relationship between the measured absorbance values at 652 nm and AA concentration was used for the detection of AA.

### Detection of the Total Antioxidant Capacity of Artificial Saliva Solutions and Biological Samples

Initially, 0.3–3.0 mM antioxidants of artificial saliva solutions were, respectively, mixed with 20 μL of Pd SAzyme solution (1.0 mg mL^−1^ in ethanol), 20 μL of TMB solution (2.0 mg mL^−1^ in DMSO), and 20 μL of H_2_O_2_ solution (10 M) and diluted to 1.0 mL by 0.2 M HAc-NaAc buffer (pH 3.5). The above solutions were incubated at 37 °C for about 5 min. Finally, the linear relationship between the measured absorbance values at 652 nm and the total concentration of antioxidants was used for the detection of the TAL. TAL in saliva was detected under the same conditions as described above. The concentrations of the samples were adjusted to meet the linear range of GSH and AA detection.

## Results and Discussion

### Synthesis and Characterization of SA-Pd/NPC

As shown in Fig. 1, the synthesis process of SA-Pd/NPC could be divided into several steps. First, silica particles in silica suspension with a diameter of about 10 nm were used as a hard template, and aniline monomers, as precursors of carbon source and nitrogen source. The aniline monomers were polymerized on the silica particles under the catalysis of APS. At the same time, silica particles with a carbon layer aggregated into a cloud shape through spontaneous self-assembly. Secondly, the salt form of polyaniline was converted into its base form in ammonia water, to facilitate the subsequent interaction between Lewis acids and bases. Thirdly, a certain amount of palladium metal salt was added to a polar aprotic solvent for Lewis acid doping. The single-site Pd could be produced by its insertion in the nitrogen caves of NPC by the Lewis acid doping strategy. As a Lewis acid, palladium could coordinate with the nitrogen atom on the polyaniline skeleton to form a polyaniline-transition metal salt complex [35, 36]. Palladium is difficult to be successfully loaded without nitrogen, which is critical in providing sites for the loading of palladium. Based on this, single-atomic palladium was introduced into the synthesized composite material by adding a palladium precursor. Next, the above materials were pyrolyzed in an inert gas at a high temperature (800 °C) for 3 h. Finally, the silicon dioxide nanoparticle template was etched by sodium hydroxide and ethanol to obtain SA-Pd/NPC.

**Fig. 2:**
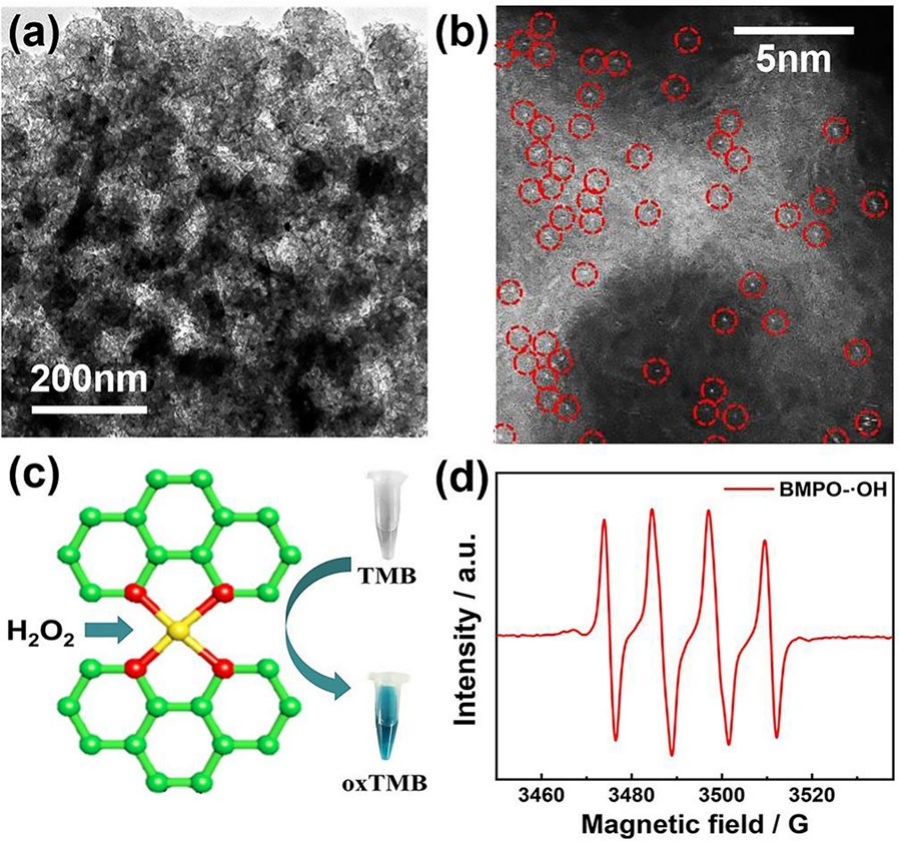
Morphological and structural characterizations of the SA-Pd/NPC sample. **a** Typical TEM image; **b** HAADF-STEM images at magnification; **c** Schematic illustration of the peroxidase-like activity of SA-Pd/NPC SAzymes; **d** EPR spectrum for the detection of •OH radicals

According to the transmission electron microscope (TEM, Fig. 2a) image, the final material had a smaller pore size than that of initial silica nanoparticles, perhaps due to the collapse of the carbon layer, which was caused by the pyrolysis of polyaniline. The finally obtained composite material was cloud-shaped and had a large number of mesopores, due to the etching of silica nanoparticles. The HAADF-STEM (Fig. 2b) image shows that the single palladium atoms were evenly distributed on the carbon matrix, and the carbon shell appeared as a "corner" shape. TMB was used as a chromogenic substrate to explore the peroxidase-like activity of SA-Pd/NPC (Fig. 2c). For the EPR experiment, BMPO was employed as the nitrogen trap for hydroxyl radicals to form BMPO-OH adducts for detection. Typically, 20 μL of BMPO (250 mM) and 20 μL of SA-Pd/NPC (1 mg·mL^−1^) were added into the ultrapure water (140 μL). Then, the 20 μL of H_2_O_2_ (10 mM) was injected in the buffer system to trigger the reaction. The results of EPR spectroscopy (Fig. 2d) show that SA-Pd/NPC could catalyze and activate H_2_O_2_ to generate hydroxyl radicals. The XRD (Additional file 1: Figure S1) image shows that there was no diffraction peak of palladium crystals. The (002) broad peak in the XRD spectrum confirms that the porous carbon was composed of small and randomly oriented graphite domains. TEM-EDS (Additional file 1: Figure S2) shows that single-atomic palladium and nitrogen were evenly distributed on the entire sample. The mass percentage of nitrogen was 11.26 wt%, and that of palladium was 0.27 wt%.

**Fig. 3:**
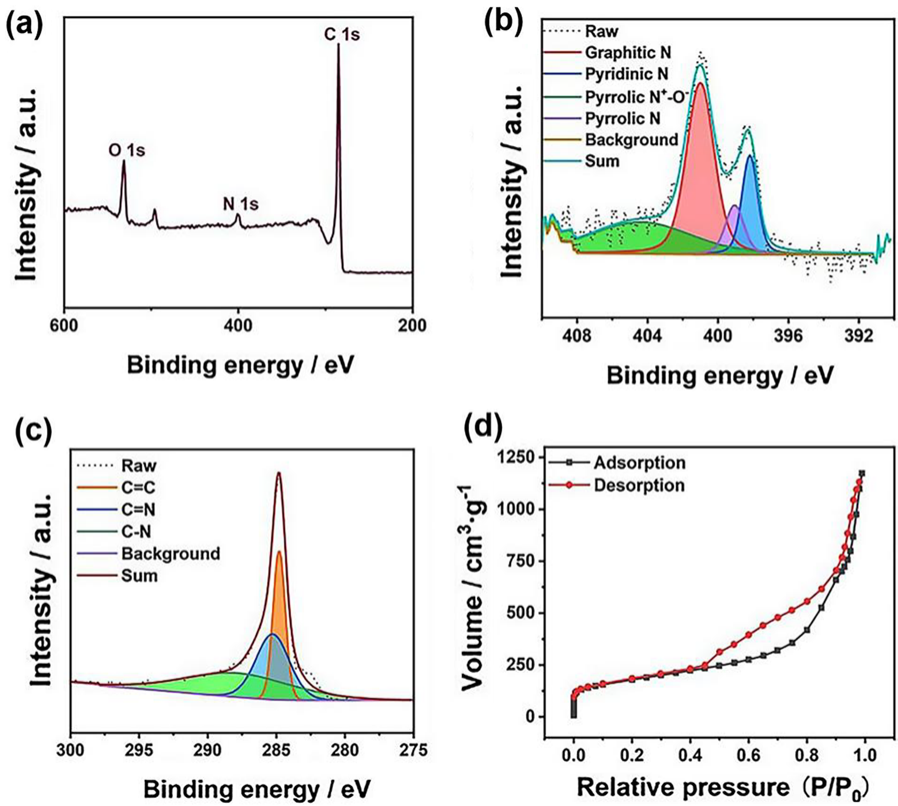
Characterizations of the structural, compositional, and electronic properties of the SA-Pd/NPC sample. **a** XPS survey spectra of the SA-Pd/NPC; **b**–**c** XPS spectra for N 1 s and C 1 s regions of SA-Pd/NPC; **d** N_2_ adsorption–desorption isotherm

The XPS survey spectrum (Fig. 3a) shows the presence of N in the carbon matrix and the doping level of nitrogen was 3.48 at.%. High-resolution XPS element-specific scanning spectra were also used to characterize the valence state of N (Fig. 3b) and C (Fig. 3c). The N 1s spectrum could be deconvoluted into four different bands at 398.1, 398.9, 400.6, and 401.4 eV, which corresponded to pyridinic, pyrrolic, graphitic, and oxidized pyridinic nitrogen, respectively [34, 35].

Characterization of the surface chemical bonding state of the as-prepared materials was achieved by FTIR. The FTIR spectrum of SA-Pd/NPC is shown in Additional file 1: Figure S3. The infrared spectrum has a strong absorption peak at 3750 cm^−1^, which is attributed to the stretching vibration of N–H, the peaks at 3000 cm^−1^ and 2800 cm^−1^ are attributed to the stretching vibration of C–H, while the peak at 1583 cm^−1^ is considered to be the C=N stretching vibration absorption peak. The peak at 1390 cm^−1^ is the characteristic peak of sp^3^C–C single bond or sp^2^C–C in the turbostratic graphite structure. And the peaks at 1273 cm^−1^ and 1124 cm^−1^ are assigned to the stretching vibration absorption peak of C–N bond; the peaks at 750 cm^−1^ and 650 cm^−1^ are assigned to the bending vibration of C–H and N–H.

**Fig. 4:**
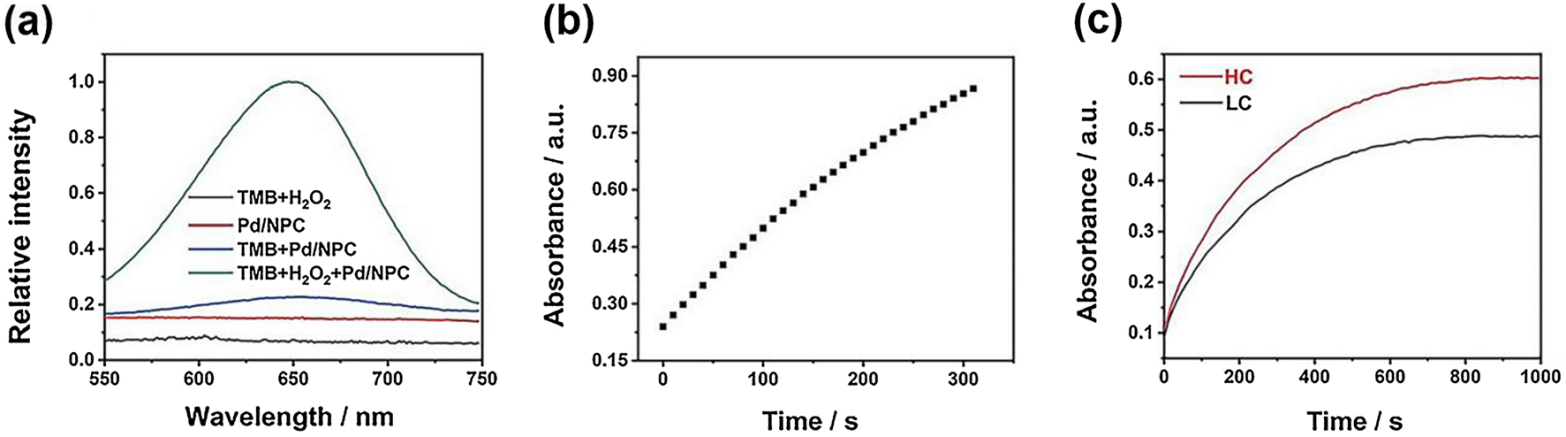
**a** Absorption spectra of the reaction products of TMB under different conditions; **b** Time-dependent absorbance changes at 652 nm of the TMB/H_2_O_2_ systems catalyzed by SA-Pd/NPC in the first 300 s; **c** Absorbance at varied Pd SAzyme concentrations (the red line represents high concentration (HC): 1 mg mL^−1^ and the black line represents low concentration (LC): 10 μg mL^−1^)

N_2_ adsorption–desorption of the prepared SA-Pd/NPC was conducted to confirm its porous structure. The N_2_ adsorption–desorption isotherm and pore size distribution curve of the sample are shown in Figs. 4d and Additional file 1: Fig. S4a. The N_2_ adsorption–desorption isotherm of Pd/NPC shows a typical type-IV curve with a hysteresis loop at a relative pressure (*P*/*P*_0_) of 0.4–1.0, which illustrates the existence of a mesoporous structure. The size of the mesopore was calculated as 3.8 nm by the Barrett–Joyner–Halenda (BJH) method, based on the desorption branch of the isotherm curve (Additional file 1: Figure S3a). Furthermore, the specific Brunauer–Emmett–Teller (BET) surface area of the SA-Pd/NPC was as high as 630.0 m^2^ g^−1^. Additionally, the SA-Pd/NPC possessed a total pore volume of 1.82 cm^3^ g^−1^. In summary, the above data show that the prepared SA-Pd/NPC had a large number of mesopores, a large surface area per unit mass, and a high pore volume. Especially, its pore size is highly conducive to further biocatalytic reactions.

The pore size distribution of the micropores was obtained by the Horvath–Kawazoe (HK) method (Additional file 1: Figure S3b). After calculation, the void volume of the micropores was 0.260 cc g^−1^, and their peak value was 0.493 nm. The total micropore volume and average micropore size showed a slit pore model with nitrogen adsorption data. The micropores were mainly created by the etching of the activator, and the attachment state of the activator was determined by the surface structures of carbon particles. Therefore, the microstructure of the carbonized material was an important factor affecting the micropore distribution.

### The Peroxidase-Like Activity of SA-Pd/NPC

Through control experiments, it was verified that SA-Pd/NPC had a peroxidase-like activity and could catalyze the oxidation of TMB in the presence of hydrogen peroxide. As shown in Fig. 4a, the absorption peak at 652 nm could be obtained only in the presence of Pd SAzyme, which indicates that TMB was oxidized to blue ox-TMB. To obtain the best reaction conditions, the effects of reaction pH and temperature were explored. As shown in Additional file 1: Figure S4a, the Pd SAzyme exhibited an outstanding catalytic efficiency under acidic reaction conditions. In particular, the maximum absorbance at 652 nm could be obtained at pH 3.5, which was selected as the general condition for subsequent reactions. As shown in Additional file 1: Figure S4b, the material displayed an excellent reactivity at temperatures lower than 45 °C. To simulate the physiological conditions of biochemical detection, 37 °C was chosen as the reaction condition for follow-up experiments. A response time curve was generated under these conditions. As shown in Figs. 4b, c, the change in absorbance in the first three hundred seconds was close to a linear increase. At about 300 s, the slope of the curve gradually decreased and reached a plateau. When the concentration of nanozyme increased, the time curve obeyed the same law. Therefore, 300 s was chosen as the reaction time.

**Fig. 5:**
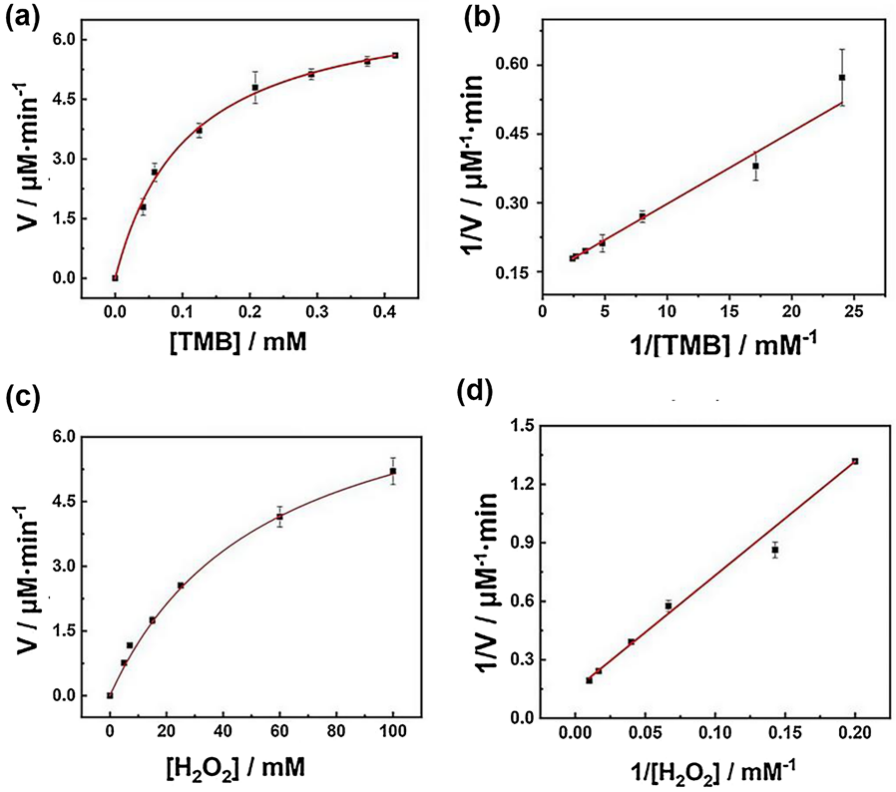
Reaction velocity under fixed H_2_O_2_ with varied concentrations of TMB (**a**) and the corresponding double-reciprocal plots of SA-Pd/NPC activity (**b**); Reaction velocity under fixed TMB with varied concentrations of H_2_O_2_ (**c**) and the corresponding double-reciprocal plots of SA-Pd/NPC activity (**d**)

The enzymatic reaction kinetic experiments were analyzed by both Michaelis–Menten curves and corresponding double-reciprocal curves. As shown in Fig. 5, TMB and H_2_O_2_ were, respectively, used as the substrate, the appearance steady-state kinetic parameters of these two curves could be acquired under a fixed concentration of H_2_O_2_ with varying concentrations of TMB, and vice versa. According to the Lineweaver–Burk plot shown in Fig. 5b, when TMB was used as a substrate, *K*_m_ = 0.1115 mM and *V*_max_ = 1.18 × 10^–7^ M s^−1^. *K*_m_ indicates the affinity between the nanozyme and the substrate. The smaller the *K*_m_ value, the greater the affinity between the enzyme and the substrate. The *K*_m_ value obtained using H_2_O_2_ as the substrate was greater than that obtained using TMB as the substrate, indicating that the Pd SAzyme had a high affinity for TMB. Moreover, as summarized in Additional file 1: Table S2, the Pd SAzyme has a much stronger affinity toward substrates and shows an excellent catalytic activity. As shown in Fig. 5b, d, the double-reciprocal curves slopes are similar, meaning that this catalytic reaction obeys a typical ping-pong mechanism, which exists in the natural enzyme-catalyzed reactions [37]. The above experimental results show that SA-Pd/NPC is highly capable of mimicking peroxidase activity. According to previous reports, the Pd SAzyme may follow a mechanism similar to that of the Fenton reaction; it catalyzes the generation of hydroxyl radicals that attach to the porous material to achieve the catalytic oxidation of the substrate [38]. According to the Sabatier principle, the excellent performance of the heterogeneous single-atomic material is derived from the adequate right force between the catalyst itself and the reaction substrate [39].

### Detection of Antioxidants Using the Pd SAzyme

**Fig. 6:**
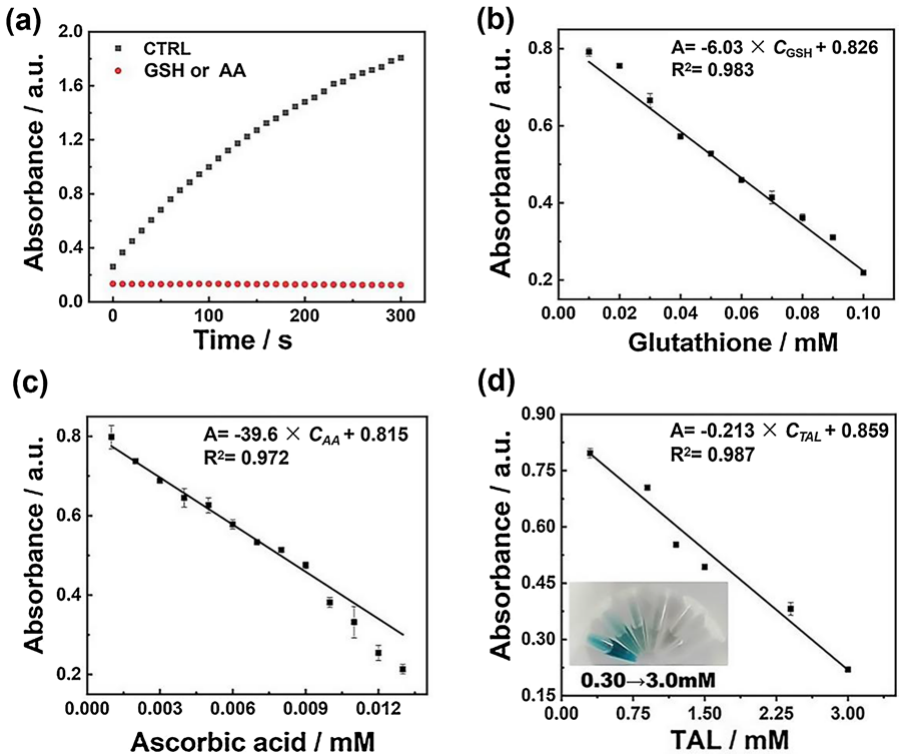
**a** Time-dependent absorbance signals at 652 nm of TMB after incubation with SA-Pd/NPC nanozymes and H_2_O_2_ in the presence of water (CTRL), a glutathione (GSH) aqueous solution, or an ascorbic acid (AA) aqueous solution. **b**–**c** Absorbance signal of the Pd/NPC nanozyme-based antioxidant assay as a function of different antioxidant molecules. **d** Total antioxidant level (TAL) analysis based on SA-Pd/NPC-simulated peroxidase for systems containing different amounts of artificial saliva. (Inset: Visual depictions of simulated saliva samples with different overall antioxidant levels under the SA-Pd/NPC peroxidase-simulated catalytic system)

As a mimic peroxidase, the SA-Pd/NPC could oxidize TMB into ox-TMB. In the presence of antioxidants, they would be prior to react with hydroxyl radicals and inhibit the TMB oxidation [40, 41]. As shown in Fig. 6a, the UV–Vis absorbance correlated with the antioxidant content in the reaction system. To verify the feasibility of the detection of antioxidants, the absorbance-time curve of the system was measured in the presence of GSH and AA, respectively. The test was performed at 37 °C, and the absorbance of the reaction system was promptly recorded after the reactant was incubated. As shown in Fig. 6a, in the control experiment, the absorbance of the solution gradually increased within 5 min and the color showed a deep blue, indicating that TMB was almost completely oxidized. However, in the presence of GSH or AA, the blue color of the system became lighter until almost invisible, suggesting a nearly total inhibition of TMB oxidation. These results mean that the developed strategy based on Pd SAzyme can detect both glutathione and ascorbic acid. Because the reaction between GSH and hydroxyl radicals obeys the hydrogen atom transfer mechanism, while AA has a single electron transfer mechanism, the common UV–Vis methods are not sensitive to detect these thiol-containing antioxidants (such as glutathione, cysteine, etc.) [42]. However, the Pd SAzyme can catalyze H_2_O_2_ to produce hydroxyl radicals, and all physiologically relevant antioxidants with both oxidation mechanisms can inhibit the TMB oxidation, so the total amount of both kinds of antioxidants can be detected by UV–Vis when this mimic peroxidase is used.

The UV–Vis absorbances at 652 nm of GSH and AA in a series of concentrations were detected to investigate their linearity. As shown in Fig. 6b, c, the UV–Vis absorbances of GSH and AA were inversely proportional to their concentration. The ideal linear responses to GSH and AA could be obtained in the concentration range of 0.01–0.10 mM and 1–13 μM (both R^2^ values were greater than 0.970), respectively. The limits of detection were 3 μM and 0.3 μM (S/N = 3), respectively. Both typical antioxidants that follow two different mechanisms to react with hydroxyl radicals could be detected with good linearity. Finally, as shown in Fig. 6d, the Pd SAzyme was used to determine the overall antioxidant level in an artificial saliva sample, which contained six kinds of physiologically relevant antioxidant components (see Additional file 1: Table S3). According to the total antioxidant content of saliva at 1.20 mM on average, the corresponding detection gradient range was selected from 0.30 to 3.0 mM [43]. A good linear detection could be achieved and the detection results were visually reflected well. As shown in Table 1, this method is more general than most TAL assays that only detect ascorbic acid [44–47].

**Table 1:** Different nanozymes were used for colorimetric detection of TAL

Nanozyme	The limit of detection (μM for AA)	The linear concentration range (μM for AA)	References
Dex-FeMnzyme	1.17	1–30	[44]
Cu-Ag/rGO	3.6	5.0–30	[45]
Lycium barbarum polysaccharide-iron (III) chelate	1.51	2–100	[46]
CMNs	30	50–5000	[47]
SA-Pd/NPC	0.3 (3 μM for GSH)	1–13 (10–100 μM for GSH)	This work

### Application to Real Saliva Samples

The biological samples testing was performed to demonstrate the potential practical application of the method. Prior to the testing, the diluted saliva samples (1:100) were prepared by dilution of pure saliva samples in ultrapure water. GSH and AA at various final concentrations (1, 3, 6, 9, 12 μM and 10, 35, 55, 80, 100 μM) were added into 100-fold diluted saliva samples. As shown in Additional file 1: Table S4, the recoveries of GSH and AA ranged from 100.58 to 105.6% and 98.08 to 112%, respectively. The results showed that the Pd SAzyme had a capacity for determining GSH and AA in real samples. It is further demonstrated that the detection method has a good anti-interference ability in a complex biological environment.

## Conclusion

In short, SA-Pd/NPC was synthesized and characterized. Aniline monomers were used as precursors to react with silica nanoparticles. The nitrogen-doped carbon shell could be generated after polymerization on the hard templates. Single palladium atoms could be anchored through Lewis acid–base action to obtain the final product. The nanozyme showed a good peroxidase-like activity by catalyzing the color reaction of TMB-H_2_O_2_. Compared to most TAL assays that used only ascorbic acid as a marker, this method had a broader response to a range of important antioxidants. It has a broad linear detection range and relatively lower detection limit, and can be applied to the detection of biological samples.

## Supplementary Information

**Additional file 1. Fig.S1.** XRD pattern of the SA-Pd/NPC. **Fig.S2** TEM-EDS spectrum of SA-Pd/NPC. **Fig.S3** FT-IR spectrum of SA-Pd/NPC. **Fig.S4** Pore size distribution. **Fig.S5** Optimization of experimental conditions. **Table S1** EDX element content of the SA-Pd/NPC. **Table S2** Comparison of the apparent kinetic parameters of different peroxidase mimics. **Table S3** The respective concentrations of physiologically relevant antioxidant components. **Table S4** Detection of AA and GSH in the human saliva samples

## Data Availability

All data supporting the conclusions of this article are included within the article and supplementary document.

## Abbreviations

SA-Pd/NPC: Single-atomic palladium-loaded nitrogen-doped porous carbon
GSH: Glutathione
AA: Ascorbic acid
AchE: Acetylcholinesterase
TAL: Total antioxidant levels
UA: Uric acid
Cys: L-cysteine
TMB: 3,3′,5,5′-Tetramethylbenzidine
APS: Ammonium persulfate
DMSO: Dimethyl sulfoxide
KSCN: Potassium thiocyanate
Hcy: DL-homocysteine
pXRD: Powder X-ray diffraction
TEM: Transmission electron microscopy
HAADF-STEM: High-angle annular dark-field scanning transmission electron microscopy
EPR: Electron paramagnetic resonance
BJH: Barrett-Joyner-Halenda
BET: Brunauer–Emmett–Teller
HK: Horvath–Kawazoe
