# Supplementary material for: Construction of Pd Single Site Anchored on Nitrogen-Doped Porous Carbon and Its Application for Total Antioxidant Level Detection
Source: Nanoscale Res Lett. 2022 May 20;17:54. doi: 10.1186/s11671-022-03693-5 (PMC9123115; doi:10.1186/s11671-022-03693-5)
Supplement: Supplementary file 1 — Additional file 1. Fig.S1. XRD pattern of the SA-Pd/NPC. Fig.S2 TEM-EDS spectrum of SA-Pd/NPC. Fig.S3 FT-IR spectrum of SA-Pd/NPC. Fig.S4 Pore size distribution. Fig.S5 Optimization of experimental conditions. Table S1 EDX element content of the SA-Pd/NPC. Table S2 Comparison of the apparent kinetic parameters of different peroxidase mimics. Table S3 The respective concentrations of physiologically relevant antioxidant components. Table S4 Detection of AA and GSH in the human saliva samples [file 11671_2022_3693_MOESM1_ESM.docx]

Supporting Information

**Construction of Pd single site anchored on nitrogen-doped porous carbon and its application for** **total antioxidant level detection**

Jingwen Zhang ^a^, Zhi Li ^a^, Hui Li ^b^, Ge Dai ^a^, Feifei Luo ^a^, Zhaohui Chu ^a^, Xing Geng ^a^, Fan Zhang ^a,^*, Qingjiang Wang ^a,^*

^a^ *School of Chemistry and Molecular Engineering, East China Normal University, 500 Dongchuan Road, Shanghai 200241, PR China*

^b^ *School of Chemistry and Chemical Engineering, Shanghai Jiaotong University, 800 Dongchuan Road, Shanghai 200240, PR China*

* Authors to whom correspondence should be addressed

E-mail address: [fzhang@chem.ecnu.edu.cn](mailto:fzhang@chem.ecnu.edu.cn) (F. Zhang); [qjwang@chem.ecnu.edu.cn](mailto:qjwang@chem.ecnu.edu.cn) (Q. J. Wang).

Tel: +86 21 54340015

**Contents**

**S1.** XRD pattern of the SA-Pd/NPC sample.

**S2.** TEM-EDS spectrum of SA-Pd/NPC complex.

**S3.** FT-IR spectrum of SA-Pd/NPC.

**S4.** Pore size distribution.

**S5.** Optimization of experimental conditions.

**S6.** Comparison of the apparent kinetic parameters of SA-Pd/NPC.

**S7.** The respective concentrations of physiologically relevant antioxidant components.

**S8.** Total Antioxidant Capacity Detection in Actual Samples.

**Reference**

**S1. XRD pattern of the SA-Pd/NPC sample.**

**
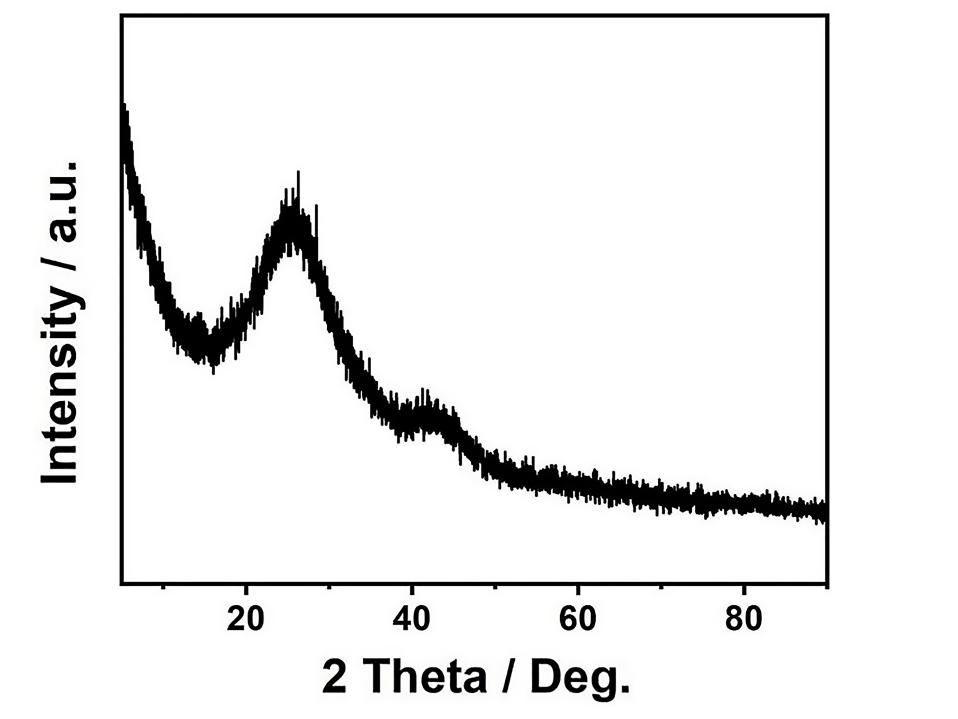
**

**Figure S1.** XRD pattern of the SA-Pd/NPC sample.

**S2. TEM-EDS spectrum of SA-Pd/NPC complex.**


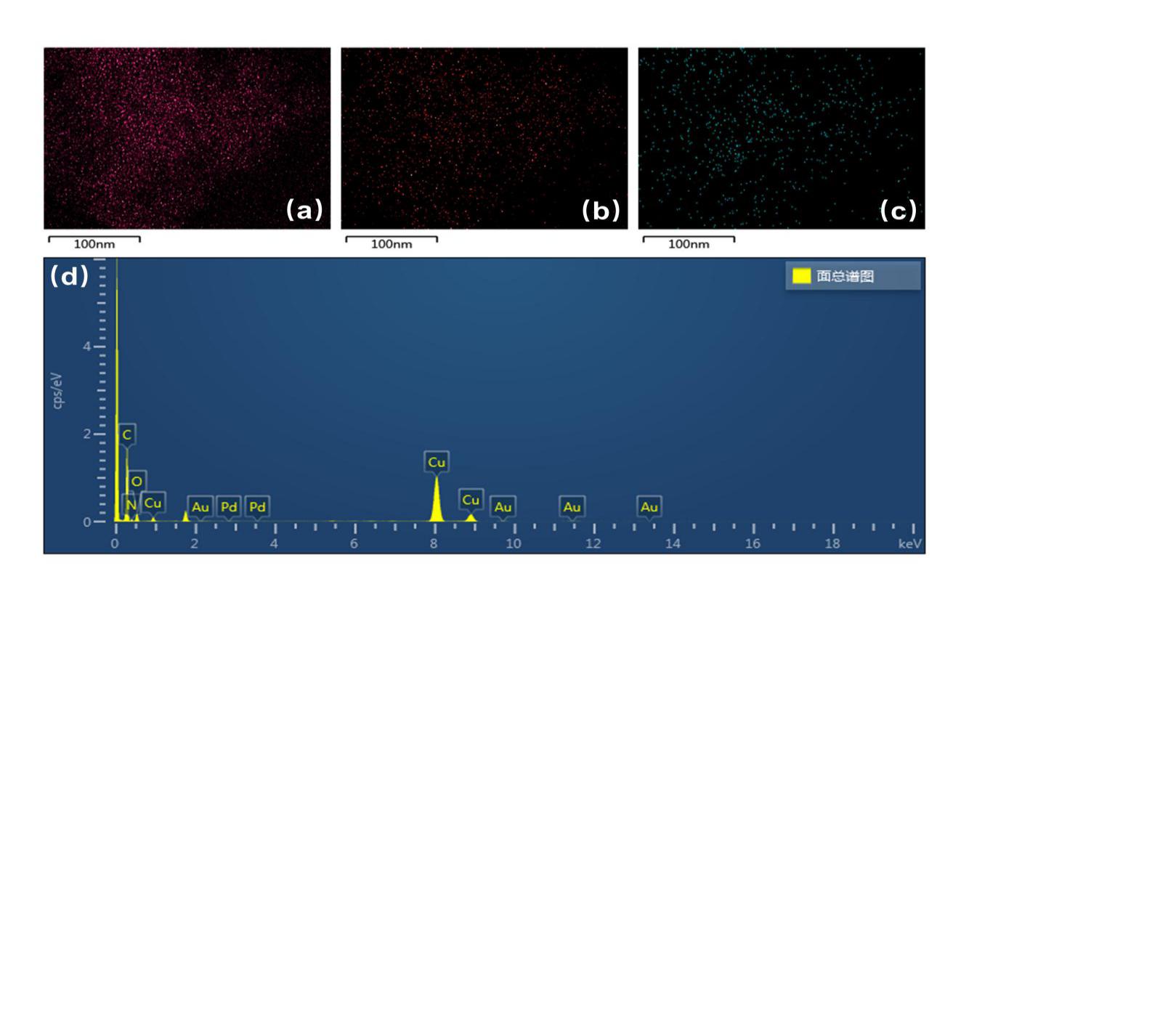


**Figure S2.** Corresponding EDS maps revealing the homogeneous distribution of (a) C, (b) N and (c) Pd, respectively. (d) TEM-EDS spectrum of SA-Pd/NPC complex.

In order to determine the catalytic mechanism of SA-Pd/NPC peroxidase-mimic activity, high resolution transmission electron microscopy (HR-TEM) and corresponding Energy dispersive X-ray spectroscopy (EDS) were recorded (Figure S2). The corresponding EDS element analysis confirmed the presence of Pd, N and C elements in the obtained SA-Pd/NPC.

**Table S1.** EDX element content of the SA-Pd/NPC.

| Element | Weight / % | Wt / % Sigma | Atomic / % |
| --- | --- | --- | --- |
| C (K) | 77.33 | 0.76 | 81.07 |
| N (K) | 11.26 | 0.36 | 10.08 |
| O (K) | 11.14 | 0.20 | 8.82 |
| Pd (K) | 0.27 | 0.05 | 0.03 |
| Totals | 100.00 |  | 100.00 |

**S3.** **FT-IR spectrum of SA-Pd/NPC.**

**
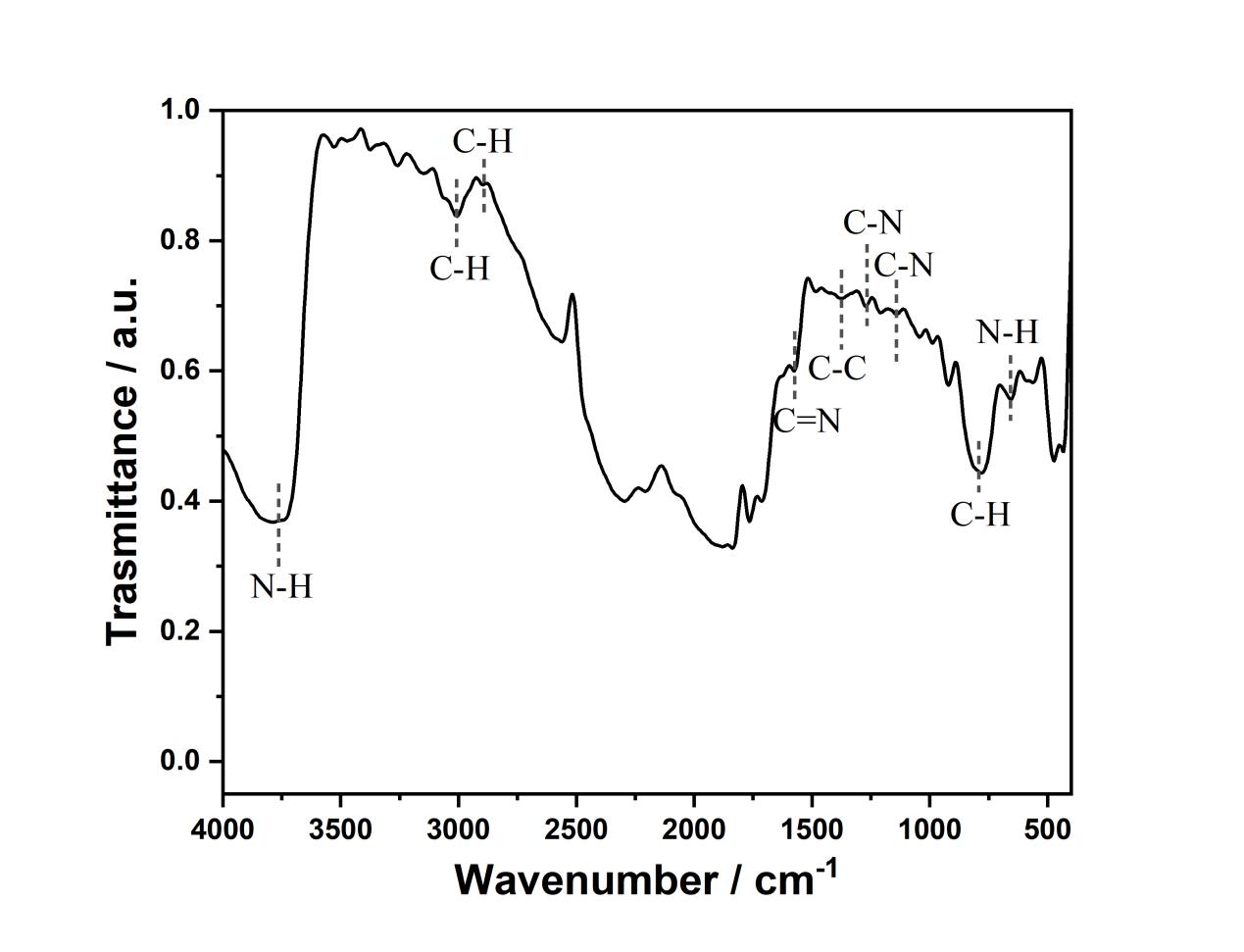
**

**Figure S3.** FT-IR spectrum of SA-Pd/NPC.

**S4. Pore size distribution.**

**
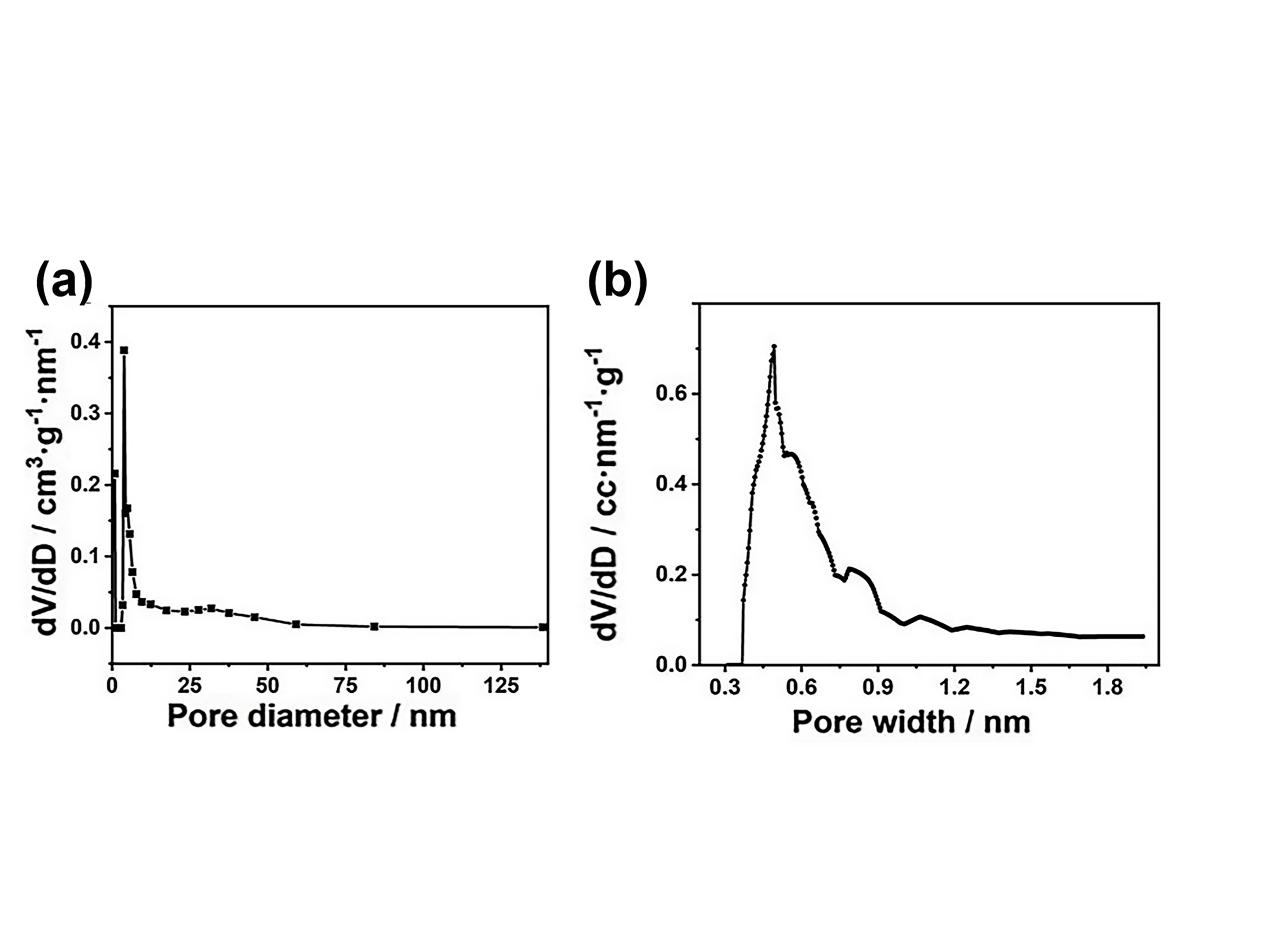
**

**Figure S4.** (a) Pore size distribution calculated from the desorption branch of the N_2_ isotherm using the BJH method; (b) HK method pore size distribution

**S5. Optimization of experimental conditions.**


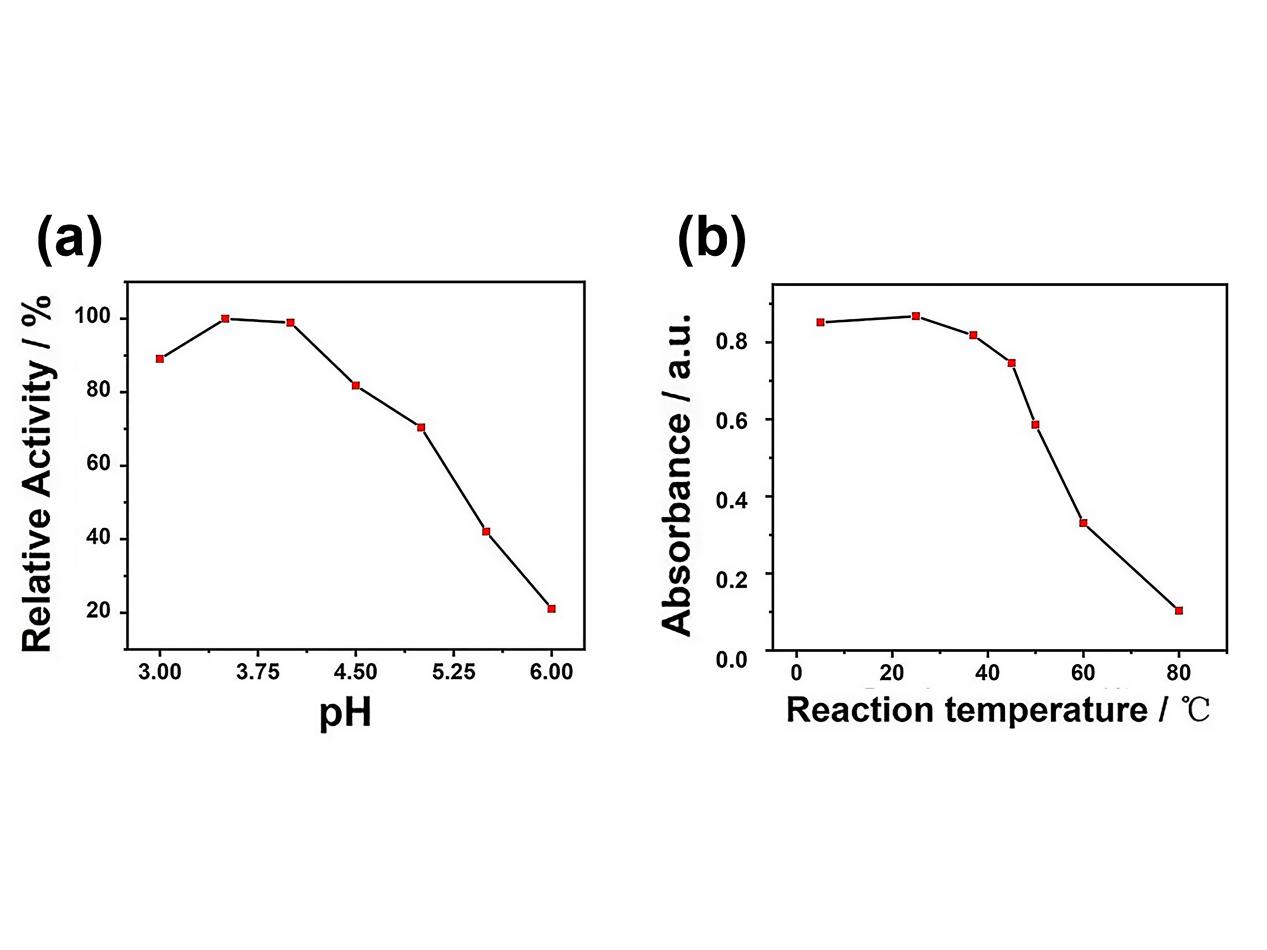


**Figure S5.** Effects of different (a) pH values of the acetate buffer and (b) incubation temperatures.

**S6. Comparison of the apparent kinetic parameters of SA-Pd/NPC.**

**Table S2.** Comparison of the apparent kinetic parameters of different peroxidase mimics.

| Material | Substance | K_m_ (mM) | V_max_ (M s^-1^) | Ref. |
| --- | --- | --- | --- | --- |
| HRP | TMB | 0.434 | 10.0×10^-8^ | 1 |
|  | H_2_O_2_ | 3.702 | 8.71×10^-8^ |  |
| Fe_3_O_4_ MNPs | TMB | 0.098 | 3.44×10^-8^ |  |
|  | H_2_O_2_ | 154 | 9.78×10^-8^ |  |
| NiC NPs | TMB | 0.123 | 6.39×10^-8^ | 2 |
|  | H_2_O_2_ | 320.5 | 6.65×10^-8^ |  |
| WS_2_ | TMB | 1.83 | 4.30×10^-8^ | 3 |
|  | H_2_O_2_ | 0.24 | 4.50×10^-8^ |  |
| Fe@CeO_2_ | TMB | 0.176 | 8.60×10^-8^ | 4 |
|  | H_2_O_2_ | 47.6 | 1.66×10^-7^ |  |
| Fe_3_O_4_@Pt | TMB | 0.147 | 7.11×10^-8^ | 5 |
|  | H_2_O_2_ | 702.6 | 7.13×10^-7^ |  |
| Pt hollow nanodendrites | TMB | 0.81 | 1.2×10^-7^ | 6 |
|  | H_2_O_2_ | 6.9 | 9.9×10^-8^ |  |
| Pt nanozyme | TMB | 0.016 | 7.18×10^-8^ | 7 |
|  | H_2_O_2_ | 165 | 11.98×10^-8^ |  |
| SA-Pd/NPC | TMB | 0.1115 | 1.18×10^-7^ | This work |
|  | H_2_O_2_ | 39.45 | 1.12×10^-7^ |  |

**S7. The respective concentrations of physiologically relevant antioxidant components.**

**Table S3.** The respective concentrations of physiologically relevant antioxidant components contained in artificial saliva.

| **TAL** | **KSCN** | **GSH** | **UA** | **AA** | **Cys** | **Hcy** | **Trolox** |
| --- | --- | --- | --- | --- | --- | --- | --- |
| **0.3** | 0.06 | 0.02 | 0.04 | 0.05 | 0.05 | 0.06 | 0.02 |
| **0.9** | 0.20 | 0.06 | 0.11 | 0.14 | 0.17 | 0.16 | 0.06 |
| **1.2** | 0.26 | 0.07 | 0.15 | 0.19 | 0.23 | 0.21 | 0.09 |
| **1.5** | 0.33 | 0.09 | 0.19 | 0.24 | 0.28 | 0.26 | 0.11 |
| **2.4** | 0.53 | 0.15 | 0.30 | 0.38 | 0.45 | 0.41 | 0.18 |
| **3.0** | 0.66 | 0.19 | 0.37 | 0.47 | 0.56 | 0.52 | 0.23 |

KSCN: thiocyanate; GSH: glutathione; UA: uric acid;

AA: ascorbic acid; Cys: cysteine; Hcy: homocysteine;

Trolox: 6-hydroxy-2,5,7,8-tetramethylchromane-2-carboxylic acid

**S8. Total Antioxidant Capacity Detection in Actual Samples.**

**Table S4.** Detection of AA and GSH in the human saliva samples.

| AA | | | GSH | | |
| --- | --- | --- | --- | --- | --- |
| Added | Found | Recovery | Added | Found | Recovery |
| (μM) | (μM) | (%) | (μM) | (μM) | (%) |
| 1 | 1.12 | 112 | 10 | 10.56 | 105.6 |
| 3 | 3.21 | 107 | 35 | 36.86 | 105.31 |
| 6 | 6.49 | 108.17 | 55 | 55.32 | 100.58 |
| 9 | 9.13 | 101.44 | 80 | 81.94 | 102.42 |
| 12 | 11.77 | 98.08 | 100 | 100.89 | 100.89 |

**Reference**

1. Gao LZ, Zhuang J, Nie L, Zhang JB, Zhang Y, Gu N, Wang TH, Feng J, Yang DL, Perrett S, Yan XY (2007) Intrinsic peroxidase-like activity of ferromagnetic nanoparticles. *Nat Nanotechnol* 2: 577–583.
2. Bao Y, Hua X, Ran H, Zeng J, Wu F (2019) Metal-doped carbon nanoparticles with intrinsic peroxidase-like activity for colorimetric detection of H_2_O_2_ and glucose. *J Mater Chem B* 7: 296–304.
3. Lin TR, Zhong LS, Song ZP, Guo LQ, Wu HY, Guo QQ, Chen Y, Fu FF, Chen GN (2014) Visual detection of blood glucose based on peroxidase-like activity of WS_2_ nanosheets. *Biosens Bioelectron* 62: 302–307.
4. Jampaiah D, Reddy TS, Kandjani AE, Selvakannan PR, Sabri YM, Coyel VE, Shukla R, Bhargava SK (2016) Fe-doped CeO_2_ nanorods for enhanced peroxidase-like activity and their application towards glucose detection. *J Mater. Chem. B* 4: 3874–3885.
5. Ma M, Xie J, Zhang Y, Chen Z, Gu N (2013) Fe_3_O_4_@Pt nanoparticles with enhanced peroxidase-like catalytic activity. *Mater Lett* 105: 36–39.
6. Ge C, Wu R, Chong Y, Fang G, Jiang X, Pan Y, Chen C, Yin JJ (2018) Synthesis of Pt Hollow Nanodendrites with Enhanced Peroxidase-Like Activity against Bacterial Infections: Implication for Wound Healing. *Adv Funct Mater* 28: 1801484.
7. Gu H, Huang Q, Zhang J, Li W, Fu Y (2020) Heparin as a bifunctional biotemplate for Pt nanocluster with exclusively peroxidase mimicking activity at near-neutral pH. *Colloids Surf* [606](https://www.sciencedirect.com/journal/colloids-and-surfaces-a-physicochemical-and-engineering-aspects/vol/606/suppl/C): 125455.
